# Formulation and Evaluation of Chondroitin Sulphate Tablets of Aceclofenac for Colon Targeted Drug Delivery

**Published:** 2012

**Authors:** Thiruganesh Ramasamy, Umadevi Subbaih Khandasamy, Suresh Shanmugam, Himabindhu Ruttala

**Affiliations:** a *Department of Bioengineering and Regenerative medicine, Utah-Inha DDS and Advanced Theapeutics Research Center, Songdo-Dong, Incheon, Republic of South Korea.*; b *Department of Pharmaceutics, Rao’s College of Pharmacy, Nellore, India*; c *Department of Pharmaceutics, School of Pharmaceutical Sciences, Vels University, Chennai, India.*; d *Department of Pharmaceutics, Pydah College of Pharmacy, Kakinada, India.*

**Keywords:** Aceclofenac, Chondroitin sulphate, HPMC K100, Eudragit coating, Colon targeting, In-vitro dissolution

## Abstract

The aim of the present study was to develop a single unit, site-specific matrix tablets of aceclofenac allowing targeted drug release in the colon with a microbially degradable polymeric carrier, chondroitin suphate (CS) and to coat the optimized batches with a pH dependent polymeric. The tablets were prepared by wet granulation method using starch mucilage as a binding agent and HPMC K-100 as a swellable polymer. Chondroitin Sulphate and drug and physical mixture were characterized by Fourier transform infrared spectroscopy (FTIR) and differential scanning calorimetry (DSC). The tablets were tested for their *in-vitro *dissolution characteristics in various simulated gastric fluids for their suitability as a colon-specific drug delivery system and also the tablets were evaluated for physicochemical properties, drug content, water percentage swelling and erosion characteristics. The dissolution data demonstrates that the 10% w/w increase in coating level of the pH dependent polymer (Eudragit L-100 and Eudragit S-100 in a ratio of 1 : 4 prevented the drug release in the simulated gastric fluid (pH 1.2-SGF) and the simulated intestinal fluid (pH 7.4-SIF). The dissolution rate of the tablet is dependent upon the concentration of Chondroitin sulphate in the simulated colonic fluid (SCF). The rapid increase in release of aceclofenac in SCF was revealed as due to the degradation of the Chondroitin sulphate membrane by bacterial enzymes. The studies confirmed that, the designed system could be used potentially as a carrier for colon delivery of aceclofenac by regulating drug release in stomach and the small intestine.

## Introduction

The oral route is considered to be most convenient for administration of drugs to patients. Oral administration of conventional dosage forms normally dissolves in the stomach fluid or intestinal fluid and absorb from these regions of the gastrointestinal tract (GIT) (1). Dosage forms that deliver drugs into the colon rather than upper GIT offers number of advantages (2, 3). Colon specific drug delivery systems are potential for not only for delivering various drugs to combat the local diseases of colon such as crohn’s disease, ulcerative colitis,constipation and colon cancer but also for delivering some drugs for the systemic absorption for treating some diseases such as rheumatoid arthritis, nocturnal asthma, hypertension which possess circadian rhythms in their symptoms (4-7).

There are several strategies being followed for targeting drugs specifically to the colon. Some of them are, pH dependent, time-controlled, pressure-controlled, and those based on biodegradable polymers (enzyme controlled) .Natural polysaccharides are now extensively used for the development of solid dosage forms for delivery of drugs to the colon. Some of the natural polysaccharides which have already been studied for their potential as colon-specific drug carrier systems are chitosan, pectin, chondroitin sulphate, cyclodextrin, dextrans, guar gum, inulin,amylose and locust bean gum (8, 9). 

The rationale for the development of a polysaccharide based delivery system for colon is the presence of large amounts of polysaccharidases in the human colon as the colon is inhabited by a large number and variety of bacteria which secrete many enzymes *e.g*. D-glucosidase, D-galactosidase, amylase, pectinase, xylanase, D-xylosidase, dextranase, *etc.* Major approaches utilizing polysaccharides for colon-specific delivery are fermentable coating of the drug core, embedding of the drug in biodegradable matrix, formulation of drug-saccharide conjugate (prodrugs) (6, 10, 11).

Chondroitin sulphate is a soluble mucopolysaccharide consisting of D-glucuronic acid linked to N-acetyl-D-galactosamide (12, 13). In the human colon, the natural sources of Chondroitin sulphate are sloughed epithelial cells and dietry meat. Chondroitin sulphate is utilized as a substrate by the *Bacteroides thetaiotaomicron *and *B*. *o*v*atus*. (14, 15). Thus Chondroitin sulphate could be used as a colonic drug carrier. Such use would depend on its persistence as a solid dosage form in the physiological environment of the stomach and small intestine. Since natural Chondroitin sulphate is readily water soluble, it might not protect its load successfully. However it would become more swellable if is mixed with a swellable polymer such as HPMC. Hence it was given a enteric coating (15).

Aceclofenac, a non steroidal anti-inflammatory drug used for the treatment of rheumatoid arthritis is selected as a model drug. Short biological half life (about 4 h) and dosing frequency more than one per day make aceclofenac an ideal candidate for sustained release. It is a newer derivative of diclofenac and has less gastrointestinal complications. The aim of the study was to design novel colon specific drug delivery systems containing Chondroitin sulphate tablets coated with pH dependent polymers (Eudragit L 100 and S 100 in the ratio of 1: 4). The goal in drug delivery research is to develop formulations to meet therapeutic needs related to particular pathological conditions. Variation of physiological and pathophysiological functions at a particular time of a day has brought a new approach to the development of drug delivery systems (16, 17). In this study we had examined the in vitro drug release of chondroitin sulphate tablets in the various simulated medium to assess its ability to release the content in colon.

**Table 1:** Composition of aceclofenac tablet.

**Ingredients**	**Quantity (mg) present in each tablet**			
	**ACST**_1_	**ACST**_2_	**ACST**_3_	**ACST**_4_
Aceclofenac	100	100	100	100
Chondroitin sulphate	50	100	150	200
HPMC K 100	25	25	25	25
Microcrystalline cellulose	25	25	25	25
Magnesium sterate (3%)	7	7	7	7
Talc (2%)	5	5	5	5
Mucilage of starch (7.5 %)	18	18	18	18

The objective of the present study was to develop a controlled release colon targeted drug delivery system of aceclofenac to approximate chronopharmacological symptoms of rheumatoid arthritis whose symptoms are most intense on awakening. The colon specific drug delivery system has a potential value when a delay in absorbtion is desired from a therapeutic point of view in the treatment of the disease rheumatoid arthritis which have peak symptoms in early morning. Research in this so called chronopharmacological field has assumed significance in developing drug delivery systems that demonstrate the importance of biological rhythms in drug therapy. Previously we successfully formulated the chitosan tablet of aceclofenac for the colonic delivery and found good release at the prerequeste time however so far there is no documented study on Chondroitin sulphate’s ability to sustain the drug release in the colon hence, in the present research we have attempted to formulate colon specific tablets of aceclofenac embedded in chondroitin suphate as a polymeric matrix and HPMC as swellable polymer which was then coated with pH dependent polymers such as Eudragit S-100 and Eudragit L-100 in a combination of 1: 4.

## Experimental

Materials used includes aceclofenac, which was kindly provided as a gift sample by Restek Pharma, Pondicherry. Chondroitin sulphate was purchased from Indian Research Products, Chennai. Eudragit S 100 and Eudragit L 100 were purchased from Loba chemicals, Mumbai. All other chemicals were of analytical grade.

*Preparation of Aceclofenac and Chondroitin sulphate tablets*

*Step-I*

Matrix tablets, each containing 100 mg of aceclofenac were prepared by wet granulation and compression using uncross-linked chondroitin sulphate as a polymer using 10 station Cadmach Mini Rotary Tablet Press supplied by Cadmach Machinary Co Pvt Ltd. The formulae of aceclofenac tablets were given in Table 1. Formulations (F_1_-F_4_) were blended and granulated with starch mucilage as a binder. The wet mass was passed through sieve number 16 (mesh size: 1000 μm) and the granules were dried at 50°C for 2-3 h. The dried granules were sieved through sieve number 25 (mesh size: 650 μm), lubricated with magnesium stearate and talc mixture and compressed on a 10 station rotary tablet punching machine, using 12 mm round slightly concave punches (17, 18). 

*Step -II*

The optimized batch of tablets were coated using a combination of Eudragit L-100 and S-100 by using a fluidized bed coating apparatus. In-process samples at various coating levels 5, 10% w/w (% coating polymeric weight gain) were taken to check the dissolution characteristics in SGF fluid. Coating solution was prepared by dissolution of 500 mg of Eudragit polymers (L-100 and S-100; 1: 1) in ethanol:acetone (2: 1) to give 10% coating. Coating was continued until there is no drug release in SGF fluid. After the coating, the tablets were gently fluidized for about 5 min after which they were air dried in an oven for 24 h at 40°C. A 10% w/w increase in the coating level was selected as an optimum coating percentage level. Then the pH dependent polymeric coated tablets were tested for drug release studies as described in the simulated gastric fluid (SGF), simulated intestinal fluid (SIF) and simulated colonic fluid (SCF) separately (19).

*Preformulation studies*

*Differential scanning calorimetry*

The DSC curves of aceclofenac, uncross linked chondroitin sulphate and mixture of aceclofenac/ chondroitin sulphate were generated by a differential scanning calorimeter (DSC 220C, SEIKO, Japan) at heating rate of 100/min from 60 to 200°C. Accurately 12 mg of sample was taken in a standard pan and placed at sample stage. Nitrogen flow was set at 50 cm^3^/min and the nitrogen flow rate to the chamber was 80 cm^3^/min. 

*Fourier transforms Infrared spectroscopy*

FT-IR spectra of aceclofenac, chondroitin sulphate and mixture of aceclofenac/chondroitin sulphate were recorded at room temperature in KBr pellets by applying 6000 kg/cm^2^ pressure by using a Shimadzu FT-IR 8300 Spectrophotometer (Shimadzu, Tokyo, Japan) in the wavelength region between 400 to 4000 cm^−1^. 

*Evaluation of granules(* 18 *-* 21 *)*

The granules were evaluated for their flow properties, the Carr index (compressibility index) and Hausner ratio. The flow rate (g/s) was calculated from the time needed for the entire sample (40 g) to empty from the funnel. Bulk density was calculated from the amount of granules poured into a 100 mL graduated cylinder up to a total volume of 50 mL while for the tap density determination, the cylinder was tapped until no measurable change in the volume was observed. The bulk density (BD) and tapped density(TD) were calculated from Equation 1 and 2.

Bulk density = Weigh of powder/ Bulk volume (Equation 1)

Tapped density = Weigh of powder/Tapped volume (Equation 2)

Based on bulk density and tap density, both the Carr Index (%) and Hausner’s ratio were calculated. 

The Carr’s index was calculated by the Equation 3:

Carr’s index (%) = [(TD-BD)*100] / TD (Equation 3)

The Hausner’s ratio was calculated by the Equation 4:

Husner’s Ratio = TD / BD (Equation 4)

Angle of repose was determined by fixed funnel method. Funnel with the end of the stem cut perpendicular to the axis of symmetry was secured with its tip at a given height (H) above a graph paper placed on a flat horizontal surface. The material was carefully poured through the funnel until the apex of the conical pile so formed just touches the tip of the funnel. The mean diameter of the base of powder cone was determined and the tangent of the angle of repose was calculated by Equation 5: 

tanα = H/R (Equation 5)

where *α* is the angle of repose.

*Evaluation of tablets (22-24)*

Prepared matrix tablets were evaluated for thickness by using digital micrometer (Mityato, Japan). Hardness of the tablet was determined by using a Monsanto hardness tester, which is expressed in kg/cm^2^. Friability of the tablet was determined using Roche Friabilator, which is expressed in percentage. Twenty tablets were initially weighed (W initial) and transferred into the friabilator. The friabilator was operated at 25 rpm per min for four minutes (per 100 revolutions). The tablets were weighed again (W final) and the percentage of friability was then calculated by using the following formula, (F = W initial-W final/W initial×100). For Weight Variation, USP 2004 procedure for uniformity of weight was followed, twenty tablets were taken and their weight was determined individually and collectively on a digital weighing balance (Shimadzu, Japan). The average weight of one tablet was determined from the collective weight.

*Drug content*

The drug content was determined by crushing and powdering five tablets from each batch separately. The amount of powder equivalent to 100 mg of the drug was weighed and dissolved in 100 mL of distlled water. After 20 min of centrifugation, aliquots of 1 mL were taken from this solution and diluted to 100 mL with water (10 μg/mL). Absorbance of the resulting solutions was measured in a UV-spectrophotometer at 275 nm. Simultaneously, a 10 μg/mL of aceclofenac standard solution was prepared in the same medium and the absorbance recorded. Content of aceclofenac was calculated (27, 28).

*In-vitro drug release studies in simulated gastric fluids*

In vitro dissolution studies for all the tablets was carried out using USP paddle method using USP XXIII dissolution apparatus at 100 rpm in 900 mL of dissolution medium (SGF) as dissolution media, maintained at 37 ± 0.5o. Five mL aliquot was withdrawn at the specified time intervals, filtered through whatmann filter paper and assayed spectrophotometrically at 275 nm using Spectrophotometer Model LUV-100A. An equal volume of fresh medium, which was prewarmed at 37°C was replaced into the dissolution media after each sampling to maintain the constant volume throughout the test. The pH of the dissolution medium was kept 1.2 for 2 h then, the pH of the dissolution medium was adjusted to 7.4 (SIF-simulated intestinal fluid) and maintained up to 24 h (29).

*In-vitro drug release study in the presence of rat caecal content (SCF-simulated colonic fluid)*

Rat caecal content was prepared by the method reported by Van den Mooter *et al*. Four albino rats of uniform body weight (150-200 g) with no prior drug treatment were used. They were weighed, maintained on normal diet, and administered 1 mL of 2% dispersion of Chondroitin sulphate in water, and this treatment was continued for 7 days for inducing the enzyme required for the degradation of Chondroitin sulphate in rats. Thirty minutes before starting the study, each rat was humanely killed and the abdomen was opened. The caecum were traced in rats, legated at both ends, dissected, and immediately transferred into phosphate buffered saline (PBS) pH 6.8, which was previously bubbled with CO_2_.

 The caecal bags were opened, the contents were weighed, homogenized and then suspended in PBS (pH 7.4) to give the desired concentration (2% w/v) of caecal content, which was used as simulated colonic fluid. The suspension was filtered through cotton wool and ultrasonicated for 10 min in an ice bath at 40% amplitude at 4°C using a probe sonicator (Soniweld, Imeco Ultrasonics, Mumbai, India) to disrupt the bacterial cell wall to release the enzyme After sonication, the mixture was centrifuged (Remi) at 2000 rpm for 20 min.

Tablets were placed in 200 mL of dissolution media (PBS, pH 7.4) containing 2% w/v rat caecal content. The experiment was performed with continuous CO_2_ supply into the dissolution medium. At different time intervals, the samples were withdrawn and replaced with fresh PBS. The experiment was continued up to 24 h. The withdrawn samples were pipetted into a series of 10 mL volumetric flasks, and volumes were made up to the mark with PBS and centrifuged. The supernatant was filtered through 0.45 µm membrane filter and the filtrate analyzed for aceclofenac content at 275 nm using UV spectrophotometer method. All the experiments were performed in triplicate (29).

*Quantification of the water uptake and erosion determination*

For conducting water uptake studies, the dissolution jars were marked with the time points of 0.5, 1, 2, up to 9 h. One tablet was placed in each dissolution jar containing 900 mL of phosphate buffer pH 7.4 buffers at 37°C ± 0.5°C, and the apparatus was run at 100 rpm using paddle. The tablets were taken out after completion of the respective stipulated time span as mentioned above and weighed, after the excess of water at the surface had been removed with filter paper. The wetted samples were then dried in an oven at 40°C up to constant weight. The increase of the weight on the tablet reflects the weight of the liquid uptake. It was estimated according to Equation 1.

Q = 100 (W w − Wi) / W w (Equation 1)

Where Q is the percentage of the liquid uptake, and Ww and Wi are the masses of the hydrated samples before drying and the initial starting dry weight, respectively.

The degree of erosion (expressed as percentage erosion of the polymer content, E) was determined using Equation 2.

E = 100 (W i − W f) W i (Equation 2)

Where Wf is the final mass of the same dried and partially eroded sample.

The entire process was repeated to get 3 values for each time point, and the average was calculated (1).

*Stability studies*

The selected formulation of tablets were stored in amber-colored glass bottles at 45°C + 75% RH for a period of 3 months as per ICH harmonized tripartite guideline for stability testing of new drug substances and products framed by Europian agency for the evaluation of medicinal products (Human medicines evaluation unit) and was observed for any change in colour, odour, and percentage drug content and cumulative drug release in various simulated GI fluids (SGF,SIFand SCF) (30, 31).

*In-vitro release kinetics*

To study the release kinetics, data obtained from *in-vitro* drug release studies were plotted in various kinetic models: zero order (Equation 3) as cumulative amount of drug released vs time, first order (Equation 4) as log cumulative percentage of drug remaining vs time, and Higuchi’s model (Equation 5) as cumulative percentage of drug released vs square root of time.

C = K_0_ t (Equation 3)

where K_0_ is the zero-order rate constant expressed in units of concentration/time and t is the time in hours. A graph of concentration vs time would yield a straight line with a slope equal to K0 and intercept the origin of the axes (32).

L o g C = L o g Co − k t / 2.303 (Equation 4)

where *C0* is the initial concentration of drug, *k* is the first order constant, and *t* is the time (33).

Q = K t 1/2 (Equation 5)

where *K* is the constant reflecting the design variables of the system and *t* is the time in hours. Hence, drug release rate is proportional to the reciprocal of the square root of time (34).

*Mechanism of drug release*

To evaluate the mechanism of drug release from chondroitin sulphate tablet, data for the first 80% of drug release were plotted in Korsmeyer *et al’s* equation (Equation 6) as log cumulative percentage of drug released vs log time, and the exponent *n* was calculated through the slope of the straight line.

Mt / M∞ = K t n (Equation 6)

where *Mt/M∞* is the fractional solute release, *t* is the release time, *K* is a kinetic constant characteristic of the drug/polymer system, and *n* is an exponent that characterizes the mechanism of release of tracers (35). For matrix tablets, if the exponent *n =* 0.45, then the drug release mechanism is Fickian diffusion, and if 0.45 < *n* < 0.89, then it is non-Fickian or anomalous diffusion. An exponent value of 0.89 is indicative of Case-II Transport or typical zero-order release (36).

*Statistical analysis*

The cumulative percentage release of aceclofenac from tablets in different medium was compared and the statistical significance was tested using student›s t-test. A value of p *< *0.05 was considered statistically significant.

## Results

*DSC studies*

**Figure 1:**
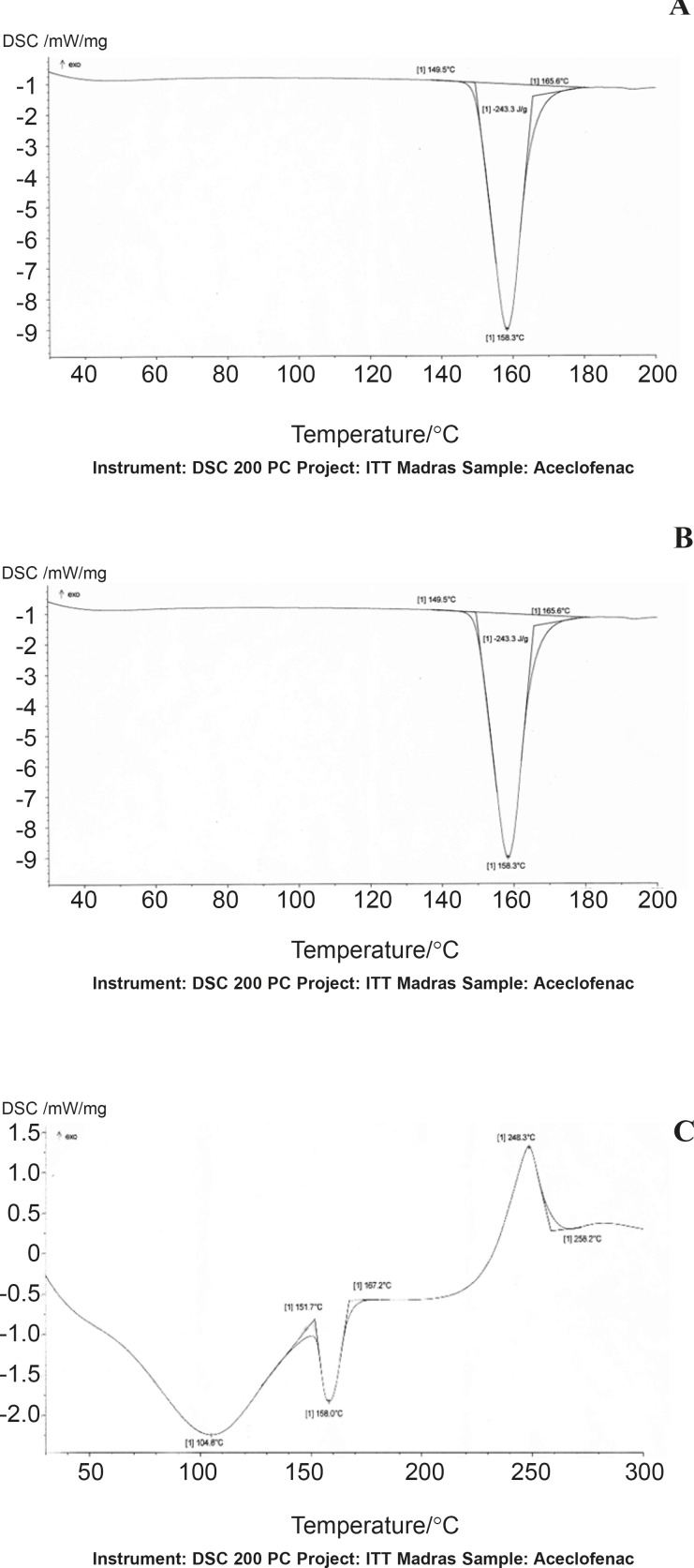
DSC thermograms of Aceclofenac (A), chondroitin sulphate (B) and physical mixture (C).

DSC thermograms of Aceclofenac, gum and mixture are depicted in (Figure 1-A, B and C), respectively. The thermogram of the pure drug exhibited a sharp endothermic peak at 158.30 °C corresponding to its melting point, while the chondroitin sulphate exhibited a broad endothermic peak at 123.80 owing to its amorphous nature, while the thermogram of physical mixture of aceclofenac and Chondroitin sulphate was 158.7°C.The DSC thermogram of the chondroitin sulphate and drug mixture showed identical peaks corresponding to pure drug indicated the absence of well defined chemical interaction between the drug and the gum.

*IR studies*

**Figure 2:**
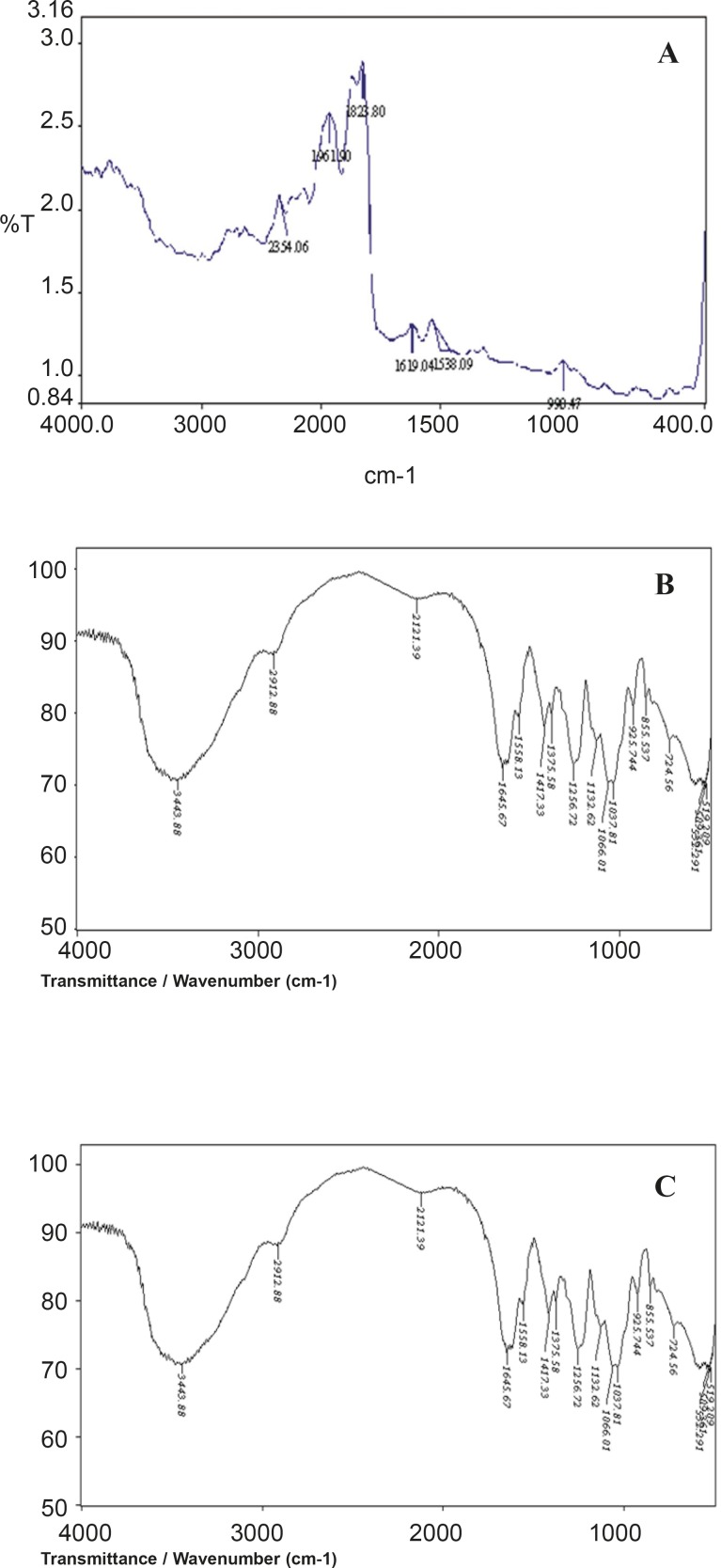
FT-IR graph of Aceclofenac (A), chondroitin sulphate (B) and physical mixture (C)

A prerequisite for the successful preparation of microparticles is the compatibility between the polymer and the drug. Drug polymer interaction when studied by FT-IR showed no drug: excipient interaction. From the FTIR spectral interpretation the following result were obtained. The FTIR of aceclofenac shows intense band at 1771.47 cm^-1^, 1716.89 cm^-1^, 1589.53 cm^-1^ and 1055.9 cm^-1^ corresponding to the functional groups C=O, COOH, NH and OH bending. The peaks observed in FTIR of physical mixture of aceclofenac and chondroitin sulphate was found to be at 1771.62 cm^-1^, 1716.76 cm^-1^, 1589.84 cm^-1^, 1055.88 cm^-1^ respectively. From the above interpretation it is understood that there is no major shifting in the frequencies of above said functional groups of aceclofenac was identified which indicates that there is no chemical interaction between aceclofenac and chondroitin sulphate which were used in the formulations. It is given in Figure 2. 

*Micromeritic properties*

The micromeritic properties of all the formulations were compared and it was found that ACST1 batch was optimal and within specified limits. The micromeritic properties of various formulations are given in Table 2.

*Physical properties*

The hardness decreased from 6.9 ± 0.12 kg/cm2 to 5.2 ± 1.06 kg/cm2, which showed that the hardness decreased gradually with the increase in chondroitin sulphate concentration and the tablet thickness increased from 2.9-4.3 mm for ACST1 to ACST4. The weight variation, thickness and percentage friability lied within the pharmacopoeial limits and were given in Table 2.

**Table 2:** Physical and Micromeritic properties of Chondroitin sulphate tablets

**Test**	**ACST**_1_	**ACST**_2_	**ACST**_3_	**ACST**_4_
Bulk density(g/cc)	0.612	0.684	0.725	0.809
Tapped density(g/cc)	0.697	0.801	0.855	0.970
Carr’s index	13.88	17.11	17.93	19.90
Hausner's ratio	1.14	1.17	1.18	1.20
Angle of repose	19°26’	20°08’	22°19’	24°18’
Weight (mg)	234 ± 1.2	285 ± 2.0	338 ± 1.2	390 ± 1.02
Drug content (%)	99.40 ± 0.20	98.2 ± 0.86	99.27 ± 0.12	98.6 ± 0.24
Hardness kg/cm2	6.9 ± 0.12	6.5 ± 0.84	5.8 ± 0.17	5.2 ± 1.06
Thickness (mm)	2.9 ± 0.6	3.2 ± 0.04	4.2 ± 0.08	4.3 ± 0.02
Friability (%)	0.17 ± 0.84	0.20 ± 0.62	0.34 ± 0.56	0.32 ± 0.48

n = 3, the values are mean ± SD

*Percentage swelling of aceclofenac and chondroitin sulfate tablets*

**Figure 3:**
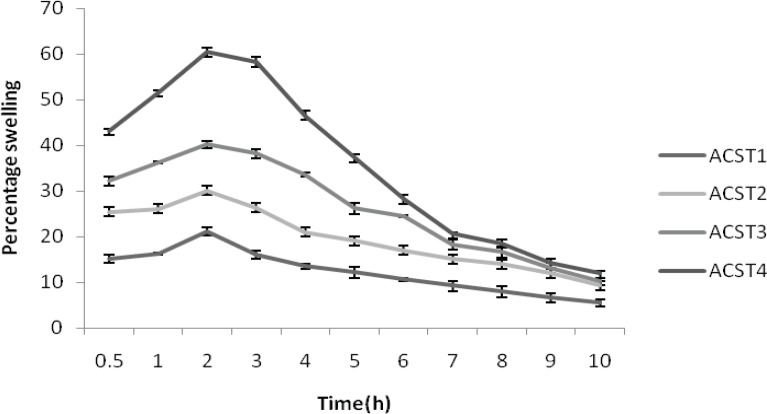
Percentage swelling of formulations (ACST1 – ACST4)

The percentage of water uptake was found to be 15.12 ± 0.27% for ACST1 and this rate increased gradually with the increase in chondroitin suphate concentration. That is 25.42 ± 0.21, 32.16 ± 0.21 , and 43.15 ± 0.47% for ACST2, ACST3 and ACST4 batch at 30 min, with the increase in polymer concentration from 22% to 51%. This trend persisted for the entire study period and the water uptake capacity decreased with the prolongation of the time and finally the value ranged from 5.48 ± 1.02%-11.98 ± 0.73% for the batch from ACST1 to ACST4. The result for percentage swelling is given in Figure 3.

*Percentage erosion*

**Figure 4:**
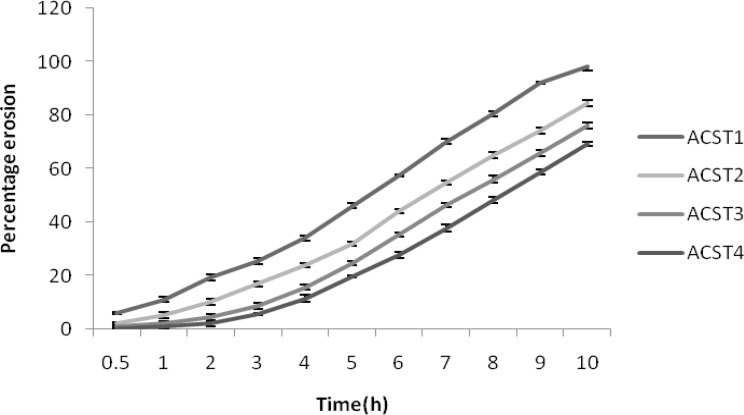
Percentage erosion of formulations (ACST1 – ACST4).

The percent erosion decreased with increase in the chondroitin sulphate concentrations. The percent erosion was 11.2 ± 1.22% for ACST1 and it decreased to 1.03 ± 0.56% for ACST4. Similarly at the 8^th^ hour, the erosion was 98.14 ± 1.65% for ACST1 and it decreased to 69.41 ± 1.34% for the ACST4. This revealed the idea that with the decrease in the HPMC: CS ratio, the release rate decreased and like wise erosion percent also decreased. The results for percentage erosion is given in Figure 4.

*In-vitro drug release studies*

*In SGF (pH 1.2 buffer)*

ACST1 tablets released about 4.12 ± 0.62 % of drug at the end of 120th min. The percentage drug release increased from ACST1 to ACST4 (*i.e*. from 4.120.62 to 10.16 ± 0.62%).

*In SIF (pH 7.4 buffer)*

**Figure 5:**
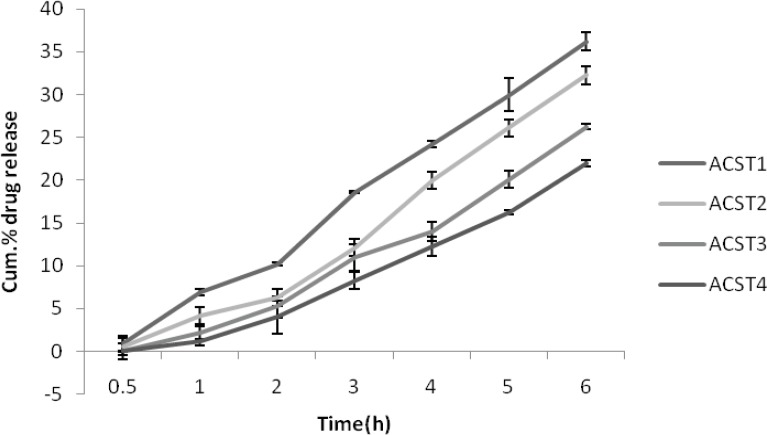
Cumulative % drug release in SIF (pH 6.8).

There was less than one percentage release for the first 2 batches (ACST1 and ACST2 ) at the end of 30 min and virtually no release took place for the next 2 (ACST3 and ACST4 ) formulations. The percentage release was 36.21 ± 0.37% for the ACST1 at the end of 6th hour which increases gradually with the increase in the chondroitin sulphate concentration, so that the final release was 22.01 ± 1.08% for ACST4 batch, which indicate that with the increase in the chondroitin sulphate concentrations, the release rate decreases.The results for percentage drug release in SIF are given in Figure 5.

*In SCF pH 7.4 buffer (with enzyme induction)*

**Figure 6:**
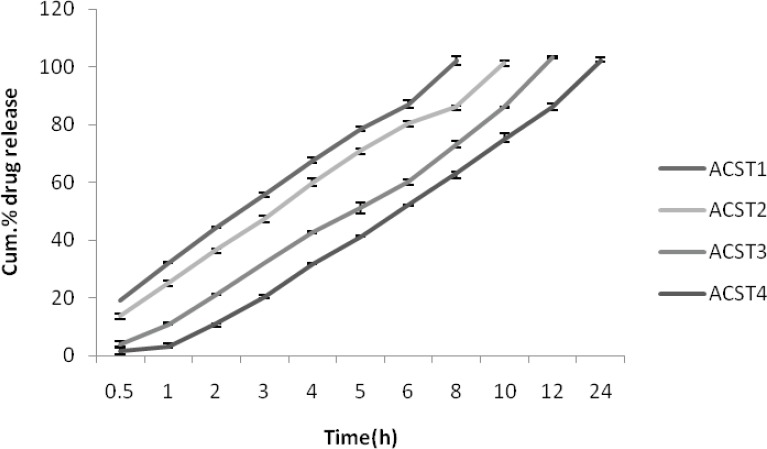
Cumulative % drug release in SCF (with enzyme induction) (pH 7.4).

As similar to that of release profile in SIF, the release profile in colonic fluid is also same where 8th hour release for ACST1 was found to be 102.24 ± 0.08% and it decreased with the increase in the chondroitin sulphate concentration and was 63.26 ± 0.18% for the ACST4 batch. The release was comparatively more than for the SIF as due to the presence of colonic enzyme which in turn helps in degradation of the polymer and leads to more release in colonic fluid. The results for percentage drug release in SCF are given in Figure 6.

*Step II*

Based on physical properties, micromeritic properties. Erosion and swelling behavior and *in-vitro* drug release characteristics, ACST1 was selected as a optimized batch from step-1 and was given pH dependent polymeric coating. The coating was done as described under the methodology. The tablets were coated to gain a weight of 10% and dried and subjected to *in-vitro* dissolution studies in simulated gastric fluids, SIF and in SCF.

*In SIF and SCF*

**Figure 7:**
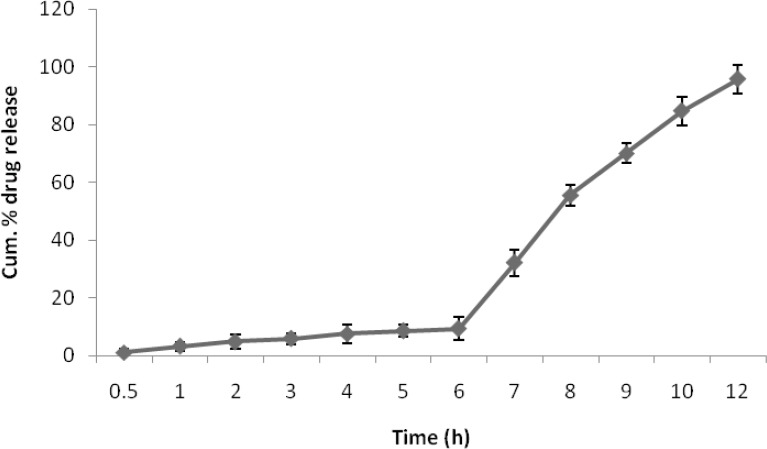
Cum. % drug release of eudragit coated E-A CST4 in SIF and SCF

There was no drug release obtained for the pH polymeric coated tablets in SGF, while drug release in SIF was very negiligible. The drug release in SCF was found to be significantly higher than in SIF(p >> 0.05). The results for percentage drug release in SIF and SCF were given in Figure 7.

*In-vitro release kinetics*

When the data were plotted according to the first order equation, all the formulation showed a fair linearity, with R2 value between (0.672-0.753), when the same data was plotted according to the zero order equation, it shown a good linearity with R2 value between (0.9801-0.9962). The treatment of the data with all the models were given in Table 3. 

**Table 3:** Release kinetics of ACST formulations

**Formulation**	**Zero order** **R** ^2^	**First order** **R** ^2^	**Higuchi** **R** ^2^	**Korsemeyer-peppas**	
				**R** ^2^	**‘n’**
ACST1	0.9962	0.753	0.998	0.994	0.45
ACST2	0.9874	0.712	0.983	0.993	0.69
ACST3	0.9815	0.752	0.981	0.984	0.56
ACST4	0.9801	0.672	0.988	0.975	0.72

*Stability studies*

The stability studies revealed that E-ACST1 formulae did not show any changes in its appearance and drug content after 6 months at an accelerated temperature and humidity condition. 

## Discussion

The colon specific drug delivery system has a potential value when a delay in absorbtion is desired from a therapeutic point of view in the treatment of the disease rheumatoid arthritis which have peak symptoms in early morning. Of the several approaches of colon specific delivery of drugs, a combination of coating with pH sensitive polymers namely Eudragit L-100 and Eudragit S-100 and embedding in biodegradable matrices (uncross linked chondroitin sulphate) has been followed for preparation of formulations. The combination of these two polymers in a various ratios makes it possible to manipulate drug release within pH range of 6.0 to 7.0. The matrices of natural polysaccharides are assumed to remain intact in the physiological environment of stomach and small intestine. But once they reach the colon they are acted upon by the bacterial polysaccharidases and result in the degradation of the matrices and release of the embedded drug. Uncross linked chondroitin sulphate have been reported to possess ideal qualities for sustained release of the drug to the targeted site (37) (colon). Colon-specific delivery of the investigational drug (aceclofenac) was aimed through single unit systems (tablets) in order to ascertain the efficacy of these formulations for delivery of drug to the colon. It has been earlier reported that polymer concentration is found to influence the release characters of the drug from the dosage form. Chondroitin sulphate in varying concentrations (22, 35, 45 and 51% w/w) were used to prepare tablets.

The method employed for tableting in this study was wet granulation and compression for which the granules should possess good flow and compacting properties. The optimum values for Carr›s index (%) and Hausner›sratio are upto 15% and less than 1.20, respectively (38). Values for angle of repose (°) less than or equal to 25 generally indicate free flowing material. By means of pilot studies it was found that Plain aceclofenac exhibited angle of repose value of 38°42’± 0.52 indicating extremely poor flow property. It was further supported by high Carr›s index value of 29.69 ± 0.27 and Hausner›s ratio of 1.44 ± 0.08. All the prepared granules Possessed good flow properties as indicated by low values of Angle of repose (19°26’±1.34–24°18’ ± 2.34), Carr›s index (13.88 ± 0.28-19.90 ± 0.09) and Hausner›s ratio (1.14 ± 0.11-1.20 ± 0.04). Since, the flow properties of the powder mixture are important for the uniformity of dose of the tablets, ACST1 was found to be good among all the formulation due its low Hausner’s ratio, compressibility index and angle of repose values.

The tablets of different batches showed varied thickness (2.9 ± 0.6 to 4.3 ± 0.02), and hardness (6.9 ± 0.12 kg/cm to 5.2 ± 1.06). The friability (0.17 ± 0.84 to 0.32 ± 0.48 %) and weight variation (%deviation: ± 2.20 to ± 4.37%) of different batches of tablets were found within the prescribed limits. The drug content was found to be uniform (> 98%) within the batches of different tablet formulations. The hardness of the tablets were decreased with the increase in chondroitin sulphate concentration where as thickness increases which will inturn increase the surface area. Thus various concentration of chondroitin sulphate did not influence the physical characteristics of the tablets, however the swelling index and the percentage erosion appears to be dependent on its concentration.

Investigation of swelling and erosion of tablets in various GI fluids is a valuable exercise to better understand the mechanisms of drug release from the matrix tablets and the relative importance of participating parameters. These data are in accord with our results as shown in Figures 1- A and B. Polymer swelling and erosion studies demonstrated a linear increase of these parameters up to 2 h. These data were fitted into the zero-order equation (r^2^ > 0.99) and the erosion parameter, with this linearity remaining during all time interval (8 h) (r^2^ > 0.99). The tablets prepared with high concentrations of chondroitin sulphate showed a lower rate of erosion and a faster rate of swelling, as compared with the tablets containing lower concentrations of chondroitin sulphate. This effect may be attributed to an increase of water uptake in the presence of a larger amount of the chondroitin sulphate and viscoelastic mass formation (39). Perhaps the viscoelastic mass formation also attributed to interaction of chondroitin sulphate with the starch mucilage due to intermolecular interaction (40) After 4 h, the linear mechanism of water uptake is altered due to the high erosion percentage of the formulations and probable change in gel structure. The swelling behavior indicated a rate at which this formulation absorbed water from dissolution media and swelled. The changes in weight, characteristic of water uptake and swelling, started from the beginning and continued until 120 min of experiment and after that it started decreasing. The percentage erosion was measured as the weight loss from matrix tablets immersed in dissolution media as a function of time. Weight loss from the tablets increased progressively with the erosion time. Polymer swelling and erosion was distinguishable among four formulations.

The evaluation of release profiles is recommended as an important tool in the development and optimization of drug formulations. The release rate of aceclofenac in first two hours in acid media was so negligible (less than 10%) for all the formulation that the result was not shown in the release curve. At this pH, aceclofenac exists in its acidic form which is well known to be practically insoluble in the stomach (40, 42). When the dissolution was changed to pH 7.4 phosphate buffer media, the drug release rate was slightly increased, possibly because the aceclofenac was partially converted to aceclofenac salt which is soluble and also because of swelling and erosion phenomenon. The release of drug in SCF is very high due to the presence of enzymes and a significant differences (p >> 0.05) was observed between different formulations formulated with varying proportions of chondroitin sulphate. The drug release from ACST1 (102.34 ± 0.36) was high, while it was low for ACST4 (63.26 ± 0.22) at 8^th^ hour of the study. The result has been reported by Jitendra *et al.* (43). The reason for this may be low concentration of chondroitin sulphate in ACST1 and high concentration in ACST4 which causes hydration of chondroitin sulphate particles in neutral medium resulted in extensive swelling. This caused initially well Separated particles to come into contact and then the Swollen particles coalesced. This resulted in a continuous viscoelastic matrix which ﬁlls the interstices, maintaining the integrity of the tablet and retarding further liquid penetration. As a result of a high swelling, chondroitin sulphate matrix tablets can sustain the drug release in SCF. In chondroitin sulphate tablets, drug release is controlled by extraction of the medicament by a simple diffusional process through an enveloping homogenous matrix and by leaching of the medicament by the bathing fluid, which is able to enter the drug matrix phase through pores, cracks and intergranular spaces. Chondroitin sulphate has a substantial ability to swell and form a hydrogel in neutral medium hence the initial drug release takes place in SIF. Where as the enteric polymers remain insoluble in the gastric pH and intestinal pH and thus controlling the release of drug within the desired range. 

The second part of the formulation focused on the pH dependent polymeric coating of the chondroitin sulphate tablets. The coating polymers were, Eudragit S-100 and Eudragit L-100, dissolves above pH 7.0, there by protecting the drug from releasing from the core before reaching the colonic region. Once the enteric coating dissolves, it is expected that drug release would then be controlled by chondroitin sulphate in the target area. Taking into account the dissolution profile of Chondroitin sulphate-aceclofenac tablets, the ACST1 was an optimized formulation as its dissolution profile was akin to the expected requirements of the study. 

The *in-vitro* release behavior of coated tablets was very dramatic. As expected, no drug release occurred at gastric pH 2.0 for 2 h. After this lag time, tablets passes through small intestine where a negligible amount of drug is released and a maximum drug release of 100.24% takes place in the colonic fluid at 8^th^ hour due to the microbial degradation of chondroitin sulphate. The mechanism of drug release from matrices containing swellable polymers is complex and not completely understood. Some systems may be classiﬁed as either purely diﬀusion or erosion controlled, while most systems exhibit a combination of these mechanisms. According to Alderman *et al.*(44), when the hydrophilic matrix system enters an *in-vitro* dissolution medium, drug particles initially pass into solution from the surface. 

The solid matrix also begins to swell as soon as hydration with solvent molecules, diffusion of the dissolved drug and erosion of viscous polymer layer into aggregates or granules and these in turn de aggregate into fine particles that also release their drug content by dissolution. Higuchi model describes the release of the drug from the matrix system through diffusion by pore formation. The release mechanism is also influenced by porosity and totuosity of the matrix (Florence *et al.*) ( 45). In this study, drug release kinetics was evaluated by fitting with different models, zero-order, first-order, Higuchi, or Krosmeyer-Peppas. According to the Table 3, it is observed that ACST1 formulation was best fitted with zero order model indicating their release kinetics is not dependent on the concentration of drug in the depot. Interestingly, release data of all the tablet formulations ﬁtted well with zero-order release model with a correlation coeﬃcient (r^2^) greater than 0.98.This is probably due to the extensive swelling of tablets. Where as matrix tablets did not show a good fit with first order model with a correlation coefficient (r^2^) between 0.67-0.75. From the correlation coefficient values (shown in Table 3), it appears that the Higuchi model seems the best-fitting model, which indicates a diffusion-controlled release.

The drug release data were ﬁtted to the power law or the Korsmeyer–Peppas equation as shown in Table 3. The Mechanism of drug release from matrices containing swellable polymers is complex and not completely understood. Some systems may be classiﬁed as either purely diﬀusion or erosion controlled, while most systems exhibit a combination of these mechanisms. In this study, the aceclofenac release, in neutral medium, from chondroitin sulphate tablets showed a good ﬁt into the Korsmeyer-Peppas equation, indicating combined eﬀect of diffusion and erosion mechanisms for drug release. As illustrated in Table 3, it exhibited a correlation coeﬃcient (r^2^) greater than 0.98 for all the formulation. In the case of matrix tablets, 0.45 < n corresponds to a Fickian diffusion mechanism and n = 0.89 indicates a purely relaxed controlled delivery which is referred to as Case II transport. Intermediate values 0.45 < n < 0.89 indicate an anomalous behavior (non-Fickian kinetics corresponding to coupled diffusion/polymer relaxation). Occasionally, values of n > 0.89 have been observed, which has been regarded as Super Case II kinetics. The calculated exponents (n) were in the range from 0.45 To 0.72, indicating an anomalous transport mechanism. The release pattern swiftly deviated from ACST1 to ACST 4. This may also be attributed to the synergistic increase in gel viscosity at the tablet periphery. This will decrease the rate of advancement of swelling front into the glassy matrix resulting in a slow diffusion of the drug. As the swelling front advances into the glassy polymer, the rubbery state, polymer (gel at the tablet periphery) which is devoid of the drug, undergoes attrition. When these two rates are equal, the diffusional path length for the drug remains constant and zero-order release will be seen. The value of r^2^ was also found to be higher for the Higuchi model. Hence the release pattern also follows the Higuchi model. The solvent molecule diffuses into the polymer creates an osmotic pressure. The initial swelling at the surface then creates additional surface for the additional penetrant and thus leads to the formation of viscoelastic mass which gives its glassy state. Moreover the accumulation of solvent around the periphery of tablet creates a solvent shock which also causes the drug release. At the end of the release process, all the polymer materials are completely dissolved in case if it follows the zero order and higuchi mechanisms. The drug release slows down with the increase in the viscosity of the gel forming polymer. The insoluble polymeric matrix of the table core of the present invention is sufficiently strong to retard the rate of release of the active ingredient or drug from the core. In addition, the tablet core provides a backbone for the outer rate controlling membrane. The polymeric matrix core prevents shrinkage and rupture of the membrane coating on exposure to gastrointestinal fluid. It also provides a tortuous path for penetrating gastrointestinal fluid, thereby keep a drug reservoir in the membrane coated polymeric matrix for a longer period of time.

The results of accelerated stability studies, carried out according to ICH guidelines, indicated thatE-ACST1 tablets did not show any changes in physical parameters (colour, friability and hardness) during the study period and the drug Content was found above 97% at the end of 180 days (0 day : 99.45 ± 0.22%; 15 days:98.26 ± 0.81%; 30 days:98.01 ± 0.16%; 60 days:97.88 ± 0.12%; 90 days:97.67 ± 0.65%; 180 days:97.01 ± 0.43%). This indicates that E-ACST1 tablet exhibited good physical stability and acceptable potency at accelerated storage condition for 6 months. 

From the overall results on the behavior of chondroitin sulphate-aceclofenac tablets, it is understood that, the drug release could be a result of the combination of hydration of chondroitin sulphate and enzymatic degradation by colonic bacterial enzymes. The present study focussed on viability of tablets of aceclofenac with uncross linked chondroitin sulphate for controlled and colon specific delivery in chronotherapy of rheumatoid arthritis where delay in delivery is preferable. A further detailed study in human subjects will through more light on their efficacy and compliance.
